# Baseline Characteristics Associated with Hypoglossal Nerve Stimulation Treatment Outcomes in Patients with Obstructive Sleep Apnea: A Systematic Review

**DOI:** 10.3390/life14091129

**Published:** 2024-09-07

**Authors:** Eldar Tukanov, Dorine Van Loo, Marijke Dieltjens, Johan Verbraecken, Olivier M. Vanderveken, Sara Op de Beeck

**Affiliations:** 1 Translational Neurosciences, Faculty of Medicine and Health Sciences, University of Antwerp, 2610 Wilrijk, Belgium; 2 Department of ENT, Head and Neck Surgery, Antwerp University Hospital, 2650 Edegem, Belgium; 3 Multidisciplinary Sleep Disorders Centre, Antwerp University Hospital, 2650 Edegem, Belgium; johan.verbraecken@uza.be; 4 Research Group LEMP, Faculty of Medicine and Health Sciences, University of Antwerp, 2610 Wilrijk, Belgium

**Keywords:** obstructive sleep apnea, OSA, hypoglossal nerve stimulation, HGNS, upper airway stimulation, prediction, association, sleep endoscopy, polysomnography, endotyping

## Abstract

Hypoglossal nerve stimulation (HGNS) has emerged as an effective treatment for obstructive sleep apnea (OSA). Identifying baseline characteristics that prospectively could predict treatment outcomes even better is crucial for optimizing patient selection and improving therapeutic success in the future. A systematic review was conducted following PRISMA guidelines. Literature searches in Medline, Web of Science, and Cochrane databases identified studies assessing baseline characteristics associated with HGNS treatment outcomes. Inclusion criteria focused on studies with adult patients diagnosed with OSA, treated with HGNS, and assessed using full-night efficacy sleep studies. Risk of bias was evaluated using the NICE tool. Twenty-six studies met the inclusion criteria. Commonly reported baseline characteristics with predictive potential included BMI, site of collapse, and various pathophysiological endotypes. Most studies used the original Sher criteria to define treatment response, though variations were noted. Results suggested that lower BMI, absence of complete concentric collapse at the palatal level, and specific pathophysiological traits were associated with better HGNS outcomes. This review identified several baseline characteristics associated with HGNS outcomes, which may guide future patient selection. Importantly, patients were already preselected for HGNS. Standardizing response criteria is recommended to enhance the evaluation and effectiveness of HGNS therapy in OSA patients.

## 1. Introduction

Obstructive sleep apnea (OSA) is a chronic respiratory sleep disorder with a high prevalence, affecting up to 49% of men and 23% of women between 39 and 75 years old [1]. OSA is characterized by repetitive narrowing (hypopnea) or complete closure (apnea) of the upper airway during sleep for at least 10 seconds [2]. These breathing interruptions lead to intermittent hypoventilation and hypercapnia. As a result, OSA is known to be associated with multiple comorbidities, such as hypertension, cardiovascular disease, type 2 diabetes, and stroke [2,3,4]. Several risk factors have been linked to OSA predisposition, including obesity, age, and anatomical features like a large neck circumference or retrognathia [5]. Men are generally more prone to OSA, though the risk for women increases post-menopause [6,7]. Lifestyle factors, including smoking and alcohol consumption, exacerbate OSA by relaxing throat muscles and increasing inflammation [7]. Recognizing these risk factors and associated comorbidities is important for the diagnosis and management of OSA.

Diagnosis is established through polysomnography (PSG), which measures various parameters throughout the night, including airflow, electroencephalography, electromyography, oxygen desaturation and heart rate. Using these measures, OSA severity is quantified by the apnea-hypopnea index (AHI), capturing the number of apneas and hypopneas per hour of sleep.

Given the comorbidities associated with OSA, efficient treatment is essential [3,4,8]. The standard treatment in clinical practice is continuous positive airway pressure (CPAP), which reopens the upper airway by acting as a pneumatic splint [9]. Alternative treatment options include mandibular advancement devices (MAD) that reopen the upper airway by protruding the mandible, positional therapy to avoid supine position, drug treatments such as acetazolamide, hypoglossal nerve stimulation treatment and other surgical treatments [10,11,12,13,14,15,16,17].

Hypoglossal nerve stimulation (HGNS) is an innovative technique that stimulates the branches of the hypoglossal nerve responsible for tongue protrusion during inspiration, maintaining an open upper airway [18]. Currently, the most commonly used HGNS device is synchronized with inspiration, using a sensing lead placed between the external and internal intercostal muscles [19]. Extensive research has proved the efficacy of this treatment in pre-selected patients [18,20,21,22]. However, despite extensive research on upfront patient selection for HGNS, approximately one-third of patients have an incomplete response [23]. Therefore, identifying baseline characteristics that are associated with successful HGNS treatment outcomes in patients with OSA is an important research focus [24].

In general, HGNS selection criteria are based on clinical parameters such as a specific range for the AHI and a maximum body mass index (BMI), and the site of collapse [25]. Natural sleep endoscopy (NSE) is the gold standard for assessing the site of collapse but is time-intensive and requires the patient to be in natural sleep without any sedatives [26,27]. Drug-induced sleep endoscopy (DISE) is a more accessible alternative used in standard clinical practice, during which sleep is induced using propofol and/or midazolam in the operating theatre [28]. Based on previous research, the presence of complete concentric collapse (CCC) at the level of the palate during DISE is a formal exclusion criterion for HGNS therapy [20]. Furthermore, four endotypic traits have been found to be associated with OSA pathophysiology: upper airway collapsibility, ventilatory control instability, muscle responsiveness, and arousal threshold [29,30]. Gold standard measurements using an overnight study with repeated pressure drops can be performed [31]. It has also been shown that these four traits can be calculated from a baseline PSG using non-invasive techniques, based on the rationale that naturally occurring apneas and hypopneas during sleep can reflect the pressure drops of the gold standard method [32]. Recently, a predictive model has been suggested to estimate the site of collapse from baseline PSG [33].

The aim of this study is to systematically review the literature on baseline characteristics of OSA patients associated with the treatment outcome of HGNS therapy in patients with OSA.

## 2. Materials and Methods

### 2.1. Data Search

From the 27th of May till the 18th of June 2024, a literature search was conducted in Medline, Web of Science and Cochrane in accordance with the PRISMA guidelines (Supplementary Table S1) [34]. The following search keywords were used: “obstructive sleep apn(o)ea”, “upper airway stimulation”, “effect” and “predictors”. The search was expanded by identifying synonyms or closely related words. Duplicates were removed. References of included articles were hand-searched to identify additional articles. The full search strategy can be found in Supplementary Table S1. Two reviewers (ET, DVL) independently assessed titles and abstracts according to in- and exclusion criteria, followed by screening of full-text articles. Each full-text article was assessed for eligibility by both reviewers. Conflicts were discussed between both reviewers. Furthermore, citation screening was performed on all included articles to search for missing articles.

### 2.2. Eligibility Criteria

Studies assessing adult human patients (18 years and older) with moderate to severe OSA, treated with respiratory-synchronized HGNS therapy and describing baseline characteristics associated with the treatment outcome of HGNS therapy were included.

Baseline characteristics associated with treatment outcome were defined as: demographic characteristics (age, sex and BMI), clinical characteristics (AHI, oxygen desaturation index (ODI), Epworth sleepiness scale (ESS) and neck circumference), pathophysiological characteristics (site of collapse, collapsibility, ventilatory control stability, muscle compensation, and arousal threshold). Other relevant parameters were also eligible if available. AHI should have been measured during a diagnostic sleep study (in-lab PSG or home sleep study), while pathophysiological characteristics should have been measured using (a combination of) drug-induced sleep endoscopy, polysomnography, or gold standard measurements. Statistical analyses assessing whether baseline characteristics were associated with HGNS treatment outcomes should have been performed. HGNS treatment outcome should have been assessed using a full-night follow-up sleep study (PSG or home sleep apnea testing (HSAT)) with the active HGNS system. If HGNS treatment outcome was only based on the results of a titration PSG, the study was excluded.

Inclusion criteria during abstract and title screening were: studies contained adult human patients with a diagnosis of OSA and patients were treated with HGNS. Inclusion criteria during full-text screening were: patients received a full-night non-titration sleep study (PSG or HSAT) at follow-up assessing the efficacy of HGNS; statistical analysis was performed to identify associations between baseline characteristics and HGNS treatment outcome.

Exclusion criteria during abstract screening were: reviews; study protocols; commentaries; single case reports; animal studies; studies only reporting patients under 18 years; study did not involve patients treated with HGNS. Full-text article exclusion was based on the presence of at least one of the following criteria: conference abstract without full text; patients did not receive a full-night efficacy sleep study at follow-up; no report of associations between baseline characteristics and HGNS treatment outcome. Language restrictions were set for English and Dutch.

### 2.3. Data Collection

A checklist of data points was used to extract data items. The checklist included: methodology (author and year of publication, trial from which patient data was extracted (if applicable), study design, data collection period), sample size (including responder and non-responder groups), hypoglossal nerve stimulation (type of device, selection criteria), time from implantation to follow-up sleep study, baseline characteristics associated with treatment outcome, statistical tests used to assess associations between baseline characteristics and treatment outcome.

### 2.4. Methodological Quality of Included Studies

To assess the methodological quality of included studies, the National Institute for Health and Clinical Excellence (NICE) quality assessment tool was used to evaluate the quality of the included studies [35]. This is a tool based on eight questions: (1) Was the case series collected in more than one center (i.e., multi-center study)? (2) Is the hypothesis/aim/objective of the study clearly described? (3) Are the inclusion and exclusion criteria (case definition) clearly reported? (4) Is there a clear definition of the outcomes reported? (5) Were data collected prospectively? (6) Is there an explicit statement that patients were recruited consecutively? (7) Are the main findings of the study clearly described? (8) Are outcomes stratified (e.g., by abnormal results, disease stage, patient characteristics)?

## 3. Results

### 3.1. Search Results and Study Characteristics

**Figure 1:**
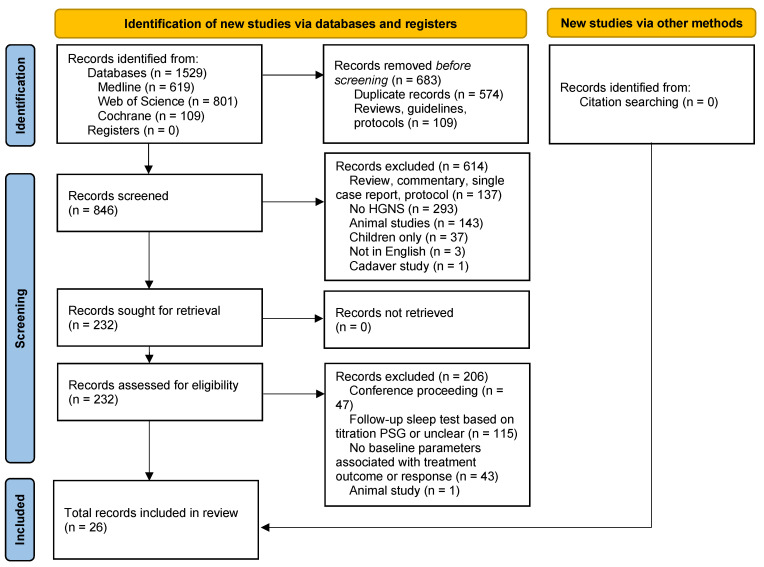
PRISMA flowchart.

The search yielded a total of 1529 results (Figure 1, PRISMA flowchart). After removal of duplicates and filtering of review, guidelines and protocols, 846 articles were screened on title and abstract. A total of 232 articles were included for full-text screening, which resulted in a final set of 26 articles that met the inclusion criteria.

An overview of all 26 articles included in this systematic review can be found in Table 1. Additional information for each article can be found in Supplementary Table S2 which includes HGNS implantation criteria and the statistical tests used to assess associations between baseline characteristics and HGNS treatment outcome. All articles required a minimum AHI of at least 15 events per hour. Out of all 26 articles, 7 articles were an analysis of the Adherence and Outcome of Upper Airway Stimulation for OSA International Registry (ADHERE), an ongoing multicenter observational study enrolling patients who received Inspire HGNS therapy (Inspire Medical Systems) to collect evidence on safety and efficacy in a standard clinical practice setting. Furthermore, four studies were a sub-analysis of the original Stimulation Therapy for Apnea Reduction (STAR) trial [18] and one study was specifically based on the German post-market study (GPMS) [21]. Most studies included patients with the Inspire upper airway stimulation device, while one study used the HGNS device by Apnex Medical (Roseville, MN, USA). Three studies had no information available on the device used. Patients were categorized as responders (R) and non-responders (NR) with response being determined using the Sher criteria (>50% decrease in AHI and AHI < 20 at follow-up) in 20 out of 24 articles, or a modified version in two other papers. Two articles did not use Sher or a modification of this definition [36]. Notably, three articles analyzed associations between baseline characteristics and treatment response using both Sher criteria and other response criteria [22,37,38].

### 3.2. Methodological Quality of Included Studies

Overall, the included articles were of medium to high quality, with all studies satisfying at least five out of eight assessment items (Table 2). The primary limiting factor in most studies (23/26) was no mentioning of consecutive patient recruitment. Furthermore, several studies were monocentric (9/26) and/or included retrospective data collection (7/26).

### 3.3. Baseline Characteristics Associated with Treatment Response

A summary of the findings on baseline characteristics associated with HGNS treatment outcome can be found in Table 3. Detailed information on each characteristic from each included article can be found in Supplementary Tables S3–S16.

#### 3.3.1. Demographic Measurements

Fourteen studies described an association between baseline age and HGNS treatment outcome, of which Heiser et al. (2019) and Withrow et al. (2019) had age as a primary outcome (Supplementary Table S3) [23,57]. Out of these fourteen studies, most studies (10/14) reported no significant difference between responders and non-responders regarding age. However, none of these studies had age as a primary outcome. Four out of fourteen studies did not show significant differences or associations. Ong et al. (2016) found that non-responders were significantly younger (*p* = 0.04) [49]. Kent et al. (2019) and Heiser et al. (2019) reported that older age was associated with greater improvement in postoperative AHI and increased treatment success respectively [23,44]. Withrow et al. (2019) indicated that both younger (<65 years) and older (≥65 years) patients had significant AHI reductions compared to baseline (*p* = 0.01), with older patients showing a larger therapeutic reduction in AHI after one year [57].

Regarding sex, ten studies described this characteristic and its association with HGNS outcome, with only Heiser et al. (2019) and Thaler et al. (2020) describing sex as a primary outcome (Supplementary Table S4) [22,57]. Heiser et al. (2019) showed that female sex was not significantly associated with a favorable treatment response (OR = 2.62, 95% CI: 0.88 to 7.78, *p* > 0.05) [23]. On the other hand, Thaler et al. (2020) found that female sex was significantly associated with a favorable treatment response (OR = 3.363, 95% CI: 1.651 to 6.848, *p* = 0.0008) [22]. When looking at the other studies, five studies out of ten (Ong et al. (2016), Op de Beeck et al. (2021), Coca et al. (2022), Wang et al. (2022), and Yu et al. (2021)), reported no significant difference between responders and non-responders (*p* > 0.05) [37,40,49,55,58]., while Kent et al. (2019) reported no significant association with AHI reduction (coefficient = 1.93, 95% CI: −0.84 to 4.70). On the other hand, Seay et al. (2020) reported a significantly lower proportion of men in the responder group, with a difference of −38.9% (95% CI: −74.2 to −3.6) [52]. Lastly, Renslo et al. (2023) reported that male gender was significantly associated with a reduction in AHI in univariable analysis (coefficient = 9.144, 95% CI: 2.803 to 15.485, *p* = 0.005), but this association did not hold in multivariable analysis which corrected for multiple covariates [50].

Sixteen studies described baseline BMI, of which three had baseline BMI as a primary outcome (Supplementary Table S5) [22,23,53]. All three studies only included patients with a BMI of 35 kg/m^2^ or lower. The first one, Suurna et al. (2021), found no significant differences in AHI reduction between patients with BMI ≤ 32 kg/m^2^ and patients with 32 < BMI ≤ 35 kg/m^2^ [53]. For the other two studies, Heiser et al. (2019) and Thaler et al. (2020), showed that lower BMI was associated with a favorable treatment response or AHI reduction [22,57]. For the other thirteen studies, five reported no significant difference in BMI between responders and non-responders (*p* > 0.05). Seay et al. (2020) also reported no significant association between BMI and treatment response (coefficient = 1.4, 95% CI: −4.8 to 2.0) [52]. On the other hand, four out of the remaining studies did report that responders had a significantly lower BMI compared to non-responders. Furthermore, two other articles reported that lower BMI was associated with a favorable treatment response or AHI reduction based on regression analyses [44,50]. A notable study was Kezirian et al. (2014), which indicated that subjects with BMI ≤ 35 kg/m² demonstrated a significant AHI reduction (*p* < 0.001), whereas those with BMI > 35 kg/m² only showed a non-significant decrease in AHI [45].

#### 3.3.2. Clinical Measurements

All studies included patients with an AHI of at least 15 events per hour. Furthermore, six studies—including the STAR trial analyses—required a maximum AHI of 50, eleven studies—including the GPMS and ADHERE analyses—required a maximum AHI of 65, and one study required a maximum AHI of 100 (Supplementary Table S2). Six articles described no maximum AHI, while two articles did not describe any selection criteria (Supplementary Table S2). Three studies had baseline AHI as a primary outcome, of which all three showed no association with treatment response (Supplementary Table S6) [22,39,57]. Most other studies (9/15) also reported no associations between baseline AHI and treatment outcome. Conversely, Kent et al. (2019)—with a mean baseline AHI of 33.8/h (SD: 15.5)—found that a higher preoperative AHI was associated with a lower postoperative AHI (coefficient: −0.74 (95% CI: −0.82 to −0.67) [44,50]. Similarly, Renslo et al. (2023)—with no baseline AHI for the total population group provided—found that a higher preoperative AHI was associated with higher AHI reduction (coefficient: 0.758 (95% CI: 0.737 to 1.093). Furthermore, Van de Heyning et al. (2012) reported that responders had significantly lower baseline AHI (*p* < 0.01) [54].

No studies had baseline ODI, ESS, apnea- or hypopnea predominance (meaning having a higher apnea index or a higher hypopnea index) or oxygen nadir as a primary outcome. Regarding ODI, two studies showed no significant difference in baseline ODI between responders and non-responders [37,40]. Steffen et al. (2018) did show that responders had a lower baseline ODI (*p* = 0.0434; OR: 0.962, 95% CI: 0.926 to 0.999) [21]. The Epworth sleepiness scale (ESS), neck circumference, and apnea or hypopnea predominance were all not associated with HGNS treatment outcome. Furthermore, Renslo et al. (2023) described that pre-operative oxygen nadir was associated with AHI reduction in multivariable analysis correcting for preoperative AHI, preoperative oxygen nadir, BMI, age, gender, and findings during palatal coupling maneuvers, but not in univariable analysis [50].

#### 3.3.3. Pathophysiological Measurements

Seven studies reported the site of collapse (palate, oropharynx, tongue base, epiglottis) and its possible associations with treatment outcome, all assessed using DISE. Only one article, Seay et al. (2020) did not have site of collapse as a primary outcome (Supplementary Table S12) [52]. Ong et al. (2016) described that an overall higher degree of collapse during DISE—meaning a higher VOTE-score (calculated as the sum of the collapse degree (2 for complete, 1 for partial, and 0 for no collapse) at each site of obstruction for a maximum score of 8)—was associated with a favorable treatment response [49]. Specifically for palatal collapse, multiple studies highlighted significant associations [20,42,49]. Vanderveken et al. (2013) reported that treatment success was achieved in 81% of patients without CCC at the palate, whereas no success was observed in patients with CCC [20]. Furthermore, patients without palatal CCC showed significant AHI improvement despite multilevel collapse at the palate and tongue base [20]. Based on the results of this study, patients with CCC are currently ineligible for HGNS therapy, therefore, in all other studies describing site of collapse, patients with CCC are excluded. Ong et al. (2016) found that non-responders had a higher proportion of complete anteroposterior (AP) or laterolateral (LL) collapse at the level of the palate compared to therapy responders (*p* = 0.01) [49]. Interestingly, one study (Kant et al. 2024) looking specifically at a group with complete AP palatal collapse and a group with AP collapse in the upper palate and CCC at the lower palate showed that both groups had a significant AHI reduction (*p* < 0.05), but no difference in response rates between both groups was found (*p* < 0.05) [42].

Regarding oropharyngeal collapse, Ong et al. (2016) observed no significant differences between responders and non-responders in the degree of oropharynx collapse (*p* > 0.05) [49]. Interestingly, Mulholland et al. (2020) reported that patients with partial collapse at the level of the oropharynx—with or without partial palatal collapse—had better treatment outcomes compared to patients with complete collapse at the level of the oropharynx—with or without complete palatal collapse [48].

For tongue base collapse, Ong et al. (2016) did not show significant differences specifically for this site, with no other articles describing this specific site of collapse [49]. In the case of epiglottis collapse, Ong et al. (2016) found that non-responders had a higher proportion of complete AP or LL collapse at the epiglottis compared with therapy responders (*p* = 0.01) [49]. Kant (2024) reported no difference in therapeutic response between groups with and without floppy epiglottis (*p* = 0.659) [43].

There were two studies that assessed collapse patterns during pre-operative DISE maneuvers and their association with HGNS treatment outcome. Mulholland et al. (2020) assessed collapse patterns during mandibular advancement, in which the mandible was advanced to two-thirds of its maximal protrusive range with a 10-mm open bite, without eliciting an arousal [48]. This study reported that patients with minimal opening of palatal and oropharyngeal collapse during mandibular advancement had a greater decrease in AHI compared to those with robust opening (*p* = 0.02) [48]. This study also showed that patients with decreased opening of the oropharynx during mandibular advancement demonstrated greater AHI improvement compared to those with increased opening (*p* = 0.03). Another study, Renslo et al. (2023), assessed palatal and tongue base collapse patterns during jaw thrust and chin lift, which are different from the maneuver used by Mulholland et al. (2020) [50]. The results of this study showed no association between response at these collapse levels during these specific maneuvers and change in AHI during HGNS therapy.

Regarding the other four pathophysiological parameters, a single sub-analysis on the STAR trial (Op de Beeck et al. 2021) reported associations with HGNS treatment outcome (Supplementary Table S13) [37]. A high arousal threshold at baseline was associated with a greater likelihood of favorable treatment response, independent of AHI, collapsibility, and other traits (OR = 6.76, 95% CI: 2.44 to 23.3, *p* = 0.001). Greater muscle compensation was found to promote success, particularly in patients with mild airway collapsibility (OR = 4.22, 95% CI: 1.70 to 12.55, *p* = 0.004). Lower loop gain was associated with better responses to HGNS, particularly in patients with milder upper airway collapsibility (OR = 0.50, 95% CI: 0.23 to 0.98, *p* = 0.056). In terms of collapsibility, the study revealed that collapsibility was more severe in responders compared to non-responders (*p* = 0.009). Notably, milder collapsibility was linked with HGNS treatment failure in patients with non-anatomical deficits, such as higher loop gain and lower arousal threshold (OR = 0.51, 95% CI: 0.24 to 1.00, *p* = 0.060). Lee et al. (2019) also described an association between the therapeutic positive airway pressure (PAP) level of prior CPAP treatment and HGNS treatment outcome, which is an indicator of upper airway collapsibility. In this study, the low PAP group (<8 cm H_2_O) achieved a significantly larger AHI reduction and better treatment response than the high PAP group (≥8 cm H_2_O) (both *p* < 0.05) (Supplementary Table S14) [38]. The baseline sleep apnea-specific hypoxic burden—a parameter associated with cardiovascular mortality and incident heart failure and defined as the sum of individual areas under the oxygen desaturation curve—did not differ between responders and non-responders in the article of Op de Beeck et al. (2021) [37,59].

#### 3.3.4. Prior Upper Airway Surgery

Regarding history of prior upper airway surgery, two articles reported no association with treatment outcome (Supplementary Table S16). Steffen et al. (2018) reported no difference in prior upper airway surgery between responders and non-responders [21]. Kezirian et al. (2019)—which also had prior upper airway surgery as a primary outcome—reported that both overall prior upper airway surgery and the specific subgroups of previous palate surgery and previous hypopharyngeal surgery were not associated with treatment response or change in AHI [46].

## 4. Discussion

This systematic review on 26 articles describes various baseline characteristics and their associations with HGNS treatment response. While the overall results were variable, findings indicated that lower BMI, absence of CCC, and specific pathophysiological traits were associated with better HGNS outcomes. However, further research is needed to clarify the specific associations between baseline characteristics and treatment outcomes.

### 4.1. Baseline Characteristics

#### 4.1.1. Demographic Measurements

Most studies reported no significant difference in age between responders and non-responders. However, when looking at articles that described baseline age as a primary outcome [22,23,57], two out of three described older age as being associated with treatment success [23,57]. This may suggest that an increase in age will result in a higher likelihood of positive treatment outcome, and that the effect of HGNS might even increase as patients get older throughout their treatment. Sex as a possible predictor showed mixed results. As OSA is multifactorial and is more prevalent in male patients, further investigation with correction for other variables is important to assess potential sex-specific responses to HGNS [60]. Furthermore, all studies had fewer female patients than male patients, which should also be taken into account during interpretation.

Regarding baseline BMI, studies generally suggested that a lower BMI results in better outcomes. Notably, the article by Van de Heyning et al. (2012) was the first study describing a relationship between BMI and treatment response [54]. It is important to note that BMI was a selection criterion for HGNS treatment in most studies. Therefore, results should be carefully interpreted as patients with a very high BMI had already been excluded.

#### 4.1.2. Clinical Measurements

Baseline AHI presented mixed findings regarding associations with HGNS treatment outcome. Some studies, such as Kent et al. (2019) and Renslo et al. (2023), suggested that higher preoperative AHI was associated with greater improvement postoperatively [44,50]. In contrast, other studies reported no significant associations. Notably, the studies that focused on baseline AHI as a primary outcome found no association with treatment outcome [22,23,39]. This indicates that while baseline AHI can be a useful predicting parameter in some cases, it may not universally predict HGNS success. Important to note, however, is that most studies have limited their patient inclusion based on the AHI. A minimum AHI of 15 events per hour or higher was mandatory for all included articles, while many articles also had a maximum AHI value between 50 and 100 events per hour for HGNS eligibility. Especially for studies based on the STAR trial, inclusion was based on an AHI between 20 and 50 events per hour [18]. Furthermore, older cohorts from the United States also had a minimum of 20 events per hour, as the FDA only lowered the minimum AHI from 20 to 15 events per hour in 2017. European cohorts usually had an AHI requirement between 15 and 65 events per hour. These limitations and differences have likely contributed to the variations in the predictive value of baseline AHI for HGNS treatment outcome.

Associations between ODI and treatment outcome were reported by three studies, yet not as a primary outcome, with only one of them showing a significant association where a lower ODI was associated with a favorable treatment response [21]. However, as the other two studies did not show any significant differences, further research would be necessary to clarify the role of baseline ODI as a predictive marker for treatment success with HGNS.

ESS, neck circumference, or apnea versus hypopnea dependency (meaning either apnea or hypopnea predominancy) were not found to be associated with HGNS treatment outcome. More studies are needed to research their effect on HGNS response. In one study, oxygen nadir showed an association with a favorable treatment response in multivariable analysis, but not during univariable analysis [50]. Therefore, oxygen nadir may play a role in predicting HGNS treatment outcome in specific patient groups. Further research is needed to consolidate this finding.

#### 4.1.3. Pathophysiological Measurements

The results indicate that the site and extent of airway collapse significantly influence HGNS treatment outcomes. CCC at the level of the palate has already been incorporated as a formal exclusion criterion in all studies based on the study of Vanderveken et al. (2013). This is an important consideration when interpreting the results of all studies conducted afterward [14,35]. Nonetheless, the results by Ong et al. (2016) suggest that other complete collapse patterns at the palate—namely complete AP and LL collapse—might be linked to non-response as well. The lack of significant differences reported by Seay et al. (2020) highlights the variability in assessing collapse severity and the need for standardized assessment protocols [37].

For oropharyngeal, tongue base and epiglottis collapse, results showed mixed findings. An interesting finding by Ong et al. (2016) was that there was no significant difference between responders and non-responders in the degree of oropharynx collapse [49]. However, a more recent article by Huyett et al. (2021) showed that complete oropharyngeal collapse was associated with lower odds of surgical response (*p* = 0.042) [61]. Nonetheless, two included studies described that complete collapse, respectively at the level of the oropharyngeal walls and the epiglottis, might be linked to worse HGNS outcomes, but more studies would be necessary to confirm this theory as the results vary [48,49].

Two studies reported associations between DISE maneuvers and HGNS outcome [48,50]. Both studies suggest that response to mandibular advancement during DISE are not associated with better treatment outcomes. This may in part be explained by the difference in mechanism; HGNS therapy contracts and protrudes the tongue muscle, while the maneuvers during DISE involve an advancement of the whole mandible. This might also be interesting when looking at patients who had MAD therapy, as this treatment device also uses mandibular advancement as the treatment mechanism. On the other hand, collapse patterns during baseline DISE without any maneuvers should be considered when screening for good candidates for HGNS.

Other pathophysiological traits, such as arousal threshold, muscle responsiveness, loop gain, and collapsibility might also play crucial roles. Important to note is that only a single article, Op de Beeck et al. (2021), described these specific traits in patients with HGNS [37]. In this study, multivariable analysis showed that a higher baseline arousal threshold was strongly associated with successful treatment outcome, and that higher muscle compensation and lower loop gain—particularly in mild collapsibility—were associated with better HGNS outcomes. Furthermore, lower collapsibility was associated with treatment failure, specifically in patients with high loop gain and low arousal threshold. On the other hand, the article by Lee et al. (2019) reported that patients who required a low PAP (<8 cm H_2_O) of prior CPAP treatment had a better treatment response compared to those who required a higher PAP (≥ 8 cm H_2_O) [38]. Importantly, patients with CCC were excluded in both articles, resulting in a selected population with favorable anatomy. As such, the findings on anatomical traits such as collapsibility should be interpreted with care.

All these findings underscore the importance of considering all these multiple pathophysiological parameters to predict treatment success effectively. In current clinical practice, site of collapse—which is usually measured using DISE—is the only pathophysiological trait that is assessed for HGNS candidacy. However, other pathophysiological traits also might play an important role as Op de Beeck et al. (2021) showed that all four traits were significantly associated with HGNS treatment outcome. Consequently, assessing these traits in HGNS candidates could improve patient selection and eventual treatment response. Traditionally, these assessments require rigorous and time-intensive procedures [31,62]. However, non-invasive techniques to estimate these traits from baseline PSG have been described, potentially removing barriers and making trait assessments more accessible in future patient selection [32,33,63,64].

#### 4.1.4. Other Measurements

Two studies specifically investigated patients who had received prior upper airway surgery and showed no significant association between prior upper airway surgery—including subgroups of previous palate surgery and previous hypopharyngeal surgery—and HGNS treatment response [21,46]. This suggests that the anatomical changes or scarring from previous surgeries did not negatively impact the ability of HGNS to improve airway patency and reduce AHI levels. As such, exclusion of patients for HGNS based solely on their surgical history would probably not be necessary. This expands the potential pool of candidates who might benefit from this therapy and underscores the strength of HGNS in managing OSA across different patient profiles.

### 4.2. Follow-Up

An important strength of this systematic review is that only studies with a full-night efficacy sleep study at follow-up were included, while studies reporting results based on titration sleep studies were excluded. Dedhia et al. (2018) argued the necessity for a full-night efficacy study in future HGNS studies, based on an analysis where titration PSG overestimated treatment response in comparison to a full-night efficacy PSG [65]. Indeed, titration studies focus on adjusting and optimizing the therapeutic settings.

By including only full-night efficacy studies, the risk of capturing an incomplete treatment effect is significantly reduced, ensuring that the outcomes reported are reflective of the treatment’s true performance over a sustained period. This approach enhances the reliability of the results, demonstrating the true benefits of HGNS in patients with OSA.

Articles with results based on a follow-up longer than 12 months were not included in this review. While a recent meta-analysis by Kim et al. (2023) found that the effects of HGNS therapy vary until 12 months after implantation and remain generally consistent between 12 and 36 months, only two articles that had a follow-up sleep study longer than 12 months after implantation described baseline characteristics associated with HGNS treatment outcome. Moreover, both articles are long-term follow-up studies of the STAR trial at three years and five years respectively [18,66,67]. Both studies—Woodson et al. (2016) and Woodson et al. (2018), had very similar results compared to Ong et al. (2016), with only differences in baseline AHI between responders and non-responders in the three-year follow-up study, and lower baseline ODI in responders at three- and five-year follow-up.

### 4.3. Limitations

The most important limitation of this systematic review is that the patients included in most studies were pre-selected for HGNS therapy based on specific criteria [18,20,54]. These criteria—which are based on the current selection criteria for patients seeking HGNS therapy—include a specific range of AHI, a maximum BMI, the absence of significant medical conditions, and since 2013, the absence of CCC at the level of the palate. Since many of these criteria inherently exclude a significant portion of the overall OSA population, particularly those with common comorbidities and pronounced risk factors, it is challenging to generalize the predictive value of baseline characteristics for HGNS treatment response across all OSA patients. This selection bias limits the applicability of our findings to the broader OSA population and may skew the perceived efficacy and predictors of HGNS. Regarding medical conditions, the inclusion criteria across various studies varied. While multiple studies specifically stated that patients with major health issues were excluded, others did not (Supplementary Table S2). This variability could potentially influence the outcomes.

The current review did not focus on cardiovascular outcomes, while this is of course an important factor to take into account regarding treatment outcome. For the current review, we opted to focus on response as defined using the AHI, as this is used in clinical practice. However, future research should focus on the cardiovascular outcomes. A recent study by Dedhia et al. (2024) involving 60 participants compared active HGNS therapy with sham treatment and found no significant differences in blood pressure after five and ten weeks [68]. These findings could suggest that HGNS therapy may have a limited impact on cardiovascular burden in the short term. However, more research is needed to evaluate the long-term effects on cardiovascular burden.

A majority of the articles, 19 out of a total of 26 papers, reported a conflict of interest (see Table 1). As stated by Crossley et al. (2021), this potential bias occurs in many, if not all, domains of research, and could indeed have influenced the reported results in one way or the other [69]. This should be taken into account during interpretation of results.

Another limitation of this systematic review is the overlap of patients in the included articles, particularly those derived from analyses of the STAR trial, the German post-market study, and the ADHERE registry. These sources represent significant datasets in the evaluation of HGNS therapy for OSA and represent 12 out of 26 included articles in this review. This overlap can introduce bias, as the same patient outcomes may be reported multiple times across different studies. It is crucial to acknowledge this limitation, as it may affect the generalizability and overall interpretation of the results. One could argue however that the findings in the different papers can stand alone as a result and should not always be conflicting with another analysis of predictors, even if there is a slight overlap in terms of the patients selected for that specific analysis. To limit this bias, whether the baseline characteristic was a primary outcome of the study was taken into consideration during the description and interpretation of results.

There is an important variation in the criteria used for defining treatment response. Most articles assessing the differences between responders and non-responders (20 out of 26) used the original Sher criteria, which require an AHI reduction of at least 50% and an AHI of less than 20 during treatment [36]. However, other studies applied variations of the Sher criteria, with some requiring an AHI under 15 instead of 20, and one article defining treatment response solely based on a 50% reduction in AHI [52]. These different definitions can influence the statistical associations between the baseline characteristics and HGNS treatment outcome. An example is Thaler et al. (2020), where baseline AHI did not predict response if response was defined using the Sher criteria [22]. However, when using other definitions of therapy response, namely AHI < 10, or 50% reduction in AHI and < 10, baseline AHI did predict response, as did sex and BMI. Consequently, this inconsistency complicates the overall interpretation of findings in this review.

In this review, only baseline characteristics measured using diagnostic tools commonly employed in standard clinical practice, such as PSG and DISE were included. However, it is important to note that four articles utilized additional diagnostic tools specifically for research purposes; These included: computed tomography (CT) and cephalometry to assess the site of airway collapse [47,51], manometry [56], or CPAP during DISE to measure palatal opening pressure, a parameter closely related to upper airway collapsibility [52]. Although these diagnostic procedures have their own limitations, they could be used as an addition to current tools or even as a stand-alone diagnostic tool in the future. Future research should aim to integrate these innovative diagnostic approaches with standard practices to enhance the assessment of obstructive sleep apnea and personalizing treatment.

Lastly, there is a difference in sleep study follow-up time. While most studies performed a follow-up sleep study at 12 months, multiple studies also reported patients who received a follow-up sleep study at a much shorter time after implantation (Table 1). This discrepancy in follow-up time might result in variable results and could possibly affect the findings of this review.

## 5. Conclusions

This systematic review described multiple baseline characteristics associated with HGNS treatment outcome in patients with OSA. Current patient selection for HGNS is based on multiple criteria, including AHI, BMI, DISE and absence of significant medical conditions. In this review, BMI, site of collapse and pathophysiological endotypes were more often described as being associated with HGNS treatment outcome. However, there is substantial variation in the results, implying that more research focusing on the predictive value of baseline characteristics for HGNS is necessary. The results of this review may serve as a reference for future research. Furthermore, standardizing response criteria is recommended to enhance the evaluation and effectiveness of HGNS therapy in OSA patients.

## Figures and Tables

**Table 1:** Study characteristics.

First Author	Year	Trial (If Applicable)	Design	Primary Outcome	Sample (R:NR)	Follow-Up Sleep Study (Months)	Baseline Characteristics	Responder Definition	Device	COI
Bosschieter [39]	2022	ADHERE	Retrospective analysis of prospective data	Change in AHI after 12 months of HGNS therapy for each group	890 (593:297)	12	AHI (severity-groups)	≥50% AHI reduction + AHI < 20/h	Inspire	Yes
Coca [40]	2022	ADHERE	Retrospective analysis of prospective data	HGNS adherence in responders and non-responders	717 (497:220)	12	Age, sex, BMI, AHI, ODI, ESS	≥50% AHI reduction + AHI < 20/h	Inspire	Yes
Gao [41]	2023	/	Retrospective case series	HGNS outcomes in apnea- and hypopnea-predominance	56	12	Apnea or hypopnea predominancy	≥50% AHI reduction + AHI < 20/h	N/A	Yes
Heiser [23]	2019	ADHERE	Retrospective analysis of prospective data	Predictors of responsiveness and adherence of therapy use	227	12	Age, sex, BMI, AHI	≥50% AHI reduction + AHI < 20/h	Inspire	Yes
Kant (a) [42]	2024	/	Retrospective	Change in AHI after 12 months of HGNS therapy for each group	56 (C-AP); 10 (AP/CCC)	12	Site of collapse	≥50% AHI reduction + AHI < 20/h	Inspire	No
Kant (b) [43]	2024	/	Retrospective	Change in AHI during HGNS therapy for groups with and without floppy epiglottis	75 (53:22)	12	Site of collapse	≥50% AHI reduction + AHI < 20/h	Inspire	No
Kent [44]	2019	STAR, GPMS, ADHERE, US cohort study	Prospective and retrospective	OSA severity at baseline associated with postoperative HGNS outcomes	584 (450:134)	12	Age, sex, BMI, AHI, neck circumference	≥50% AHI reduction + AHI < 20/h	Inspire	Yes
Kezirian [45]	2014	/	Prospective	Change in AHI after 12 months of HGNS therapy	31 (17:14)	12	BMI	≥50% AHI reduction + AHI < 20/h	Apnex	Yes
Kezirian [46]	2019	ADHERE	Retrospective analysis of prospective data	% responders at final visit for groups with prior upper airway surgery	147 (116:31)	12	Prior upper airway surgery	≥50% AHI reduction + AHI <20/h	Inspire	Yes
Lee [38]	2019	/	Pro- and retrospective	Change in AHI during HGNS therapy for low and high PAP group	56 (31:25)	>6	Therapeutic PAP level	≥50% AHI reduction + AHI < 20/h (+ analyses on AHI < 10/h and < 5/h)	Inspire	No
Lee [47]	2021	/	Prospective and retrospective	Radiographic predictors of HGNS response during cephalometry	51 (24:27)	3–12	Age, BMI, AHI	≥50% AHI reduction + AHI < 20/h	Inspire	No
Mulholland [48]	2020	/	Prospective	Change in palate and oropharynx during mandibular advancement	46	N/A	Site of collapse	N/A	Inspire	No
Ong [49]	2016	STAR	Retrospective analysis of prospective data	Collapse patterns during baseline DISE predictive of HGNS response	124(84:40)	12	Age, sex, BMI, AHI, site of collapse, neck size	≥50% AHI reduction + AHI < 20/h	Inspire	Yes
Op de Beeck [37]	2021	STAR	Retrospective analysis of prospective data	Pathophysiological mechanisms underlying favorable versus incomplete responses to HGNS therapy	91 (53:38)	12	Age, sex, BMI, AHI, ODI, arousal threshold, collapsibility, muscle responsiveness, loop gain, SASHB	≥50% AHI reduction + AHI < 10/h (+ sensitivity analyses on other definitions)	Inspire	Yes
Renslo [50]	2023	/	Retrospective	Maneuvers during DISE predictive of HGNS outcome	171 (61:110)	N/A	Age, gender, AHI, oxygen nadir, site of collapse	≥50% AHI reduction + AHI < 15/h	N/A	No
Schwab [51]	2018	/	Prospective	Anatomic predictors (site of collapse) of HGNS response	13 (7:6)	12	Age, BMI, AHI	≥50% AHI reduction + AHI < 20/h	Inspire	Yes
Seay [52]	2020	/	Prospective	Therapeutic nasal PAP levels at the soft palate during DISE, predictive of HGNS response	27 (18:9)	>3	Age, sex, BMI, AHI, site of collapse	≥50% AHI reduction	Inspire	No
Steffen [21]	2018	GPMS	Prospective	Change in AHI, ESS and FOSQ after 12 months of HGNS therapy	56 (41:15)	12	Age, BMI, AHI, ODI, ESS, neck circumference, prior upper airway surgery	≥50% AHI reduction + AHI < 20/h	Inspire	Yes
Suurna [53]	2021	ADHERE	Retrospective analysis of prospective data	Change in AHI and ESS after 12 months of HGNS therapy, specifically in BMI ≤ 32 and between 32 and 35	535	12	BMI	≥50% AHI reduction + AHI < 20/h	Inspire	Yes
Thaler [22]	2020	ADHERE	Retrospective analysis of prospective data	Change in AHI after 12 months of HGNS therapy and predictors	382 (265:117)	12	Age, sex, BMI, AHI	≥50% AHI reduction + AHI <20/h (+ sensitivity analyses on ≥50% AHI reduction + AHI < 10/h, and AHI < 10)	Inspire	Yes
Vanderveken [20]	2013	/	Prospective	Collapse patterns during baseline DISE predictive of HGNS response	21 (13:8)	6	Site of collapse	≥50% AHI reduction + AHI < 20/h	Inspire	Yes
Van de Heyning [54]	2012	/	Prospective	Change in AHI and ODI after 6 months of HGNS therapy	20 (6:14)	6	AHI, BMI, ESS	≥50% AHI reduction + AHI < 20/h	Inspire	Yes
Wang [55]	2022	/	Retrospective	Data from intra-operative tongue muscle activation associated with post-operative HGNS response	46 (28:18)	6-12	Age, sex, BMI	≥50% AHI reduction + AHI < 20/h	Inspire	Yes
Wirth [56]	2022	/	Prospective	Manometric sites of collapse predictive of HGNS response	26 (11:15)	3-12	AHI	≥50% AHI reduction + AHI < 15/h	N/A	Yes
Withrow [57]	2019	ADHERE	Retrospective analysis of prospective data	Change in AHI during HGNS therapy for old and young group	250	12	Age	N/A	Inspire	Yes
Yu [58]	2021	STAR	Retrospective analysis of prospective data	Change in AHI after 12 months of HGNS therapy	105 (72:33)	12	Age, sex, BMI, AHI, ESS, neck circumference	≥50% AHI reduction + AHI < 20/h	Inspire	Yes

R (responders); NR (non-responders); HGNS (hypoglossal nerve stimulation); DISE (drug-induced sleep endoscopy); AHI (apnea-hypopnea index); BMI (body mass index); ODI (oxygen desaturation index); ESS (Epworth sleepiness scale); PAP (positive airway pressure); SASHB (sleep apnea-specific hypoxic burden); N/A (not available). COI (conflict of interest): either funding by a company/manufacturer of HGNS, and/or consultancy for a company/manufacturer of HGNS by one or more of the authors.

**Table 2:** Methodological quality of included studies.

First Author	Quality Assessment of Included Studies							
	1	2	3	4	5	6	7	8
Bosschieter 2022 [39]	**Yes**	**Yes**	**Yes**	**Yes**	**Yes**	No	**Yes**	**Yes**
Coca 2022 [40]	**Yes**	**Yes**	**Yes**	**Yes**	**Yes**	No	**Yes**	**Yes**
Gao 2023 [41]	No	**Yes**	**Yes**	**Yes**	No	No	**Yes**	**Yes**
Heiser 2019 [23]	**Yes**	**Yes**	**Yes**	**Yes**	**Yes**	No	**Yes**	**Yes**
Kant (a) 2024 [42]	No	**Yes**	**Yes**	**Yes**	No	No	**Yes**	**Yes**
Kant (b) 2024 [43]	No	**Yes**	**Yes**	**Yes**	No	No	**Yes**	**Yes**
Kent 2019 [44]	**Yes**	**Yes**	**Yes**	**Yes**	**Yes**	No	**Yes**	**Yes**
Kezirian 2014 [45]	**Yes**	**Yes**	**Yes**	**Yes**	**Yes**	No	**Yes**	**Yes**
Kezirian 2019 [46]	**Yes**	**Yes**	**Yes**	**Yes**	**Yes**	No	**Yes**	**Yes**
Lee 2019 [38]	**Yes**	**Yes**	**Yes**	**Yes**	No	No	**Yes**	**Yes**
Lee 2021 [47]	No	**Yes**	**Yes**	**Yes**	No	No	**Yes**	**Yes**
Mulholland 2020 [48]	No	**Yes**	**Yes**	**Yes**	**Yes**	No	**Yes**	**Yes**
Ong 2016 [49]	**Yes**	**Yes**	**Yes**	**Yes**	**Yes**	No	**Yes**	**Yes**
Op de Beeck 2021 [37]	**Yes**	**Yes**	**Yes**	**Yes**	**Yes**	No	**Yes**	**Yes**
Renslo 2023 [50]	No	**Yes**	**Yes**	**Yes**	No	No	**Yes**	**Yes**
Schwab 2018 [51]	**Yes**	**Yes**	**Yes**	**Yes**	**Yes**	No	**Yes**	**Yes**
Seay 2020 [52]	No	**Yes**	**Yes**	**Yes**	**Yes**	**Yes**	**Yes**	**Yes**
Steffen 2018 [21]	**Yes**	**Yes**	**Yes**	**Yes**	**Yes**	**Yes**	**Yes**	**Yes**
Suurna 2021 [53]	**Yes**	**Yes**	**Yes**	**Yes**	**Yes**	No	**Yes**	**Yes**
Thaler 2020 [22]	**Yes**	**Yes**	**Yes**	**Yes**	**Yes**	No	**Yes**	**Yes**
Vanderveken 2013 [20]	**Yes**	**Yes**	**Yes**	**Yes**	**Yes**	No	**Yes**	**Yes**
Van de Heyning 2012 [54]	**Yes**	**Yes**	**Yes**	**Yes**	**Yes**	**Yes**	**Yes**	**Yes**
Wang 2022 [55]	No	**Yes**	**Yes**	**Yes**	No	No	**Yes**	**Yes**
Wirth 2022 [56]	No	**Yes**	**Yes**	**Yes**	**Yes**	No	**Yes**	**Yes**
Withrow 2019 [57]	**Yes**	**Yes**	**Yes**	**Yes**	**Yes**	No	**Yes**	**Yes**
Yu 2021 [58]	**Yes**	**Yes**	**Yes**	**Yes**	**Yes**	No	**Yes**	**Yes**

Assessment was conducted based on following questions: (1) Case series collected in more than one center (i.e., multicenter study)? (2) Is the hypothesis/aim/objective of the study clearly described? (3) Are the inclusion and exclusion criteria (case definition) clearly reported? (4) Is there a clear definition of the outcomes reported? (5) Were data collected prospectively? (6) Is there an explicit statement that patients were recruited consecutively? (7) Are the main findings of the study clearly described? (8) Are outcomes stratified (e.g., by disease stage, abnormal test results, patient characteristics)?

**Table 3:** Associations between baseline characteristics and HGNS treatment outcome.

Baseline Characteristic	Association	# of Studies	References
**Demographic**			
Age	Older age was associated with AHI reduction or treatment response	1	[23]
	Older patients (≥65 years) had a larger AHI reduction than younger patients (<65 years)	1	[57]
	No association between age and treatment outcome	1	[22]
	Older age was associated with AHI reduction or treatment response	2	[44,49]
	No association between age and treatment outcome	9	[21,37,40,47,50,51,52,55,58]
Sex	Female sex was associated with AHI reduction or treatment response	1	[22]
	No association between sex and treatment outcome	1	[23]
	No association between sex and treatment outcome	6	[37,40,44,49,55,58]
	Female sex was associated with AHI reduction or treatment response	1	[52]
	Male gender was associated with AHI reduction in univariable analysis, but not in multivariable analysis	1	[50]
BMI	Lower baseline BMI was associated with increased AHI reduction or treatment response	2	[22,23]
	No difference in AHI reduction between groups with BMI ≤ 32 kg/m^2^ and 32 < BMI ≤ 35 kg/m^2^	1	[53]
	No association between baseline BMI and treatment outcome	6	[21,47,49,51,52,58]
	Lower baseline BMI was associated with increased AHI reduction or treatment response	6	[37,40,44,50,54,55]
	Non-significant AHI reduction in group with BMI > 35 kg/m^2^, but significant AHI reduction in group with BMI ≤ 35 kg/m^2^	1	[45]
**Clinical**			
AHI	No association between baseline AHI and treatment outcome	3	[22,23,39]
	No association between baseline AHI and treatment outcome	9	[21,37,40,47,49,51,52,56,58]
	Higher baseline AHI was associated with increased AHI reduction or treatment response	2	[44,50]
	Lower baseline AHI was associated with increased treatment response	1	[54]
ODI	No association between baseline ODI and treatment outcome	2	[37,40]
	Lower baseline ODI was associated with treatment response	1	[21]
ESS	No association between ESS and treatment response	5	[21,40,52,54,58]
Neck circumference	No association between neck circumference and treatment response	4	[21,44,49,58]
Apnea- or hypopnea-dependent	No association between apnea or hypopnea dependency (meaning either apnea or hypopnea predominancy) and treatment response	1	[41]
Oxygen nadir	Oxygen nadir was associated with AHI reduction in multivariable analysis, but not in univariable analysis	1	[50]
**Pathophysiological**			
Site of collapse	No association between degree of oropharyngeal or tongue base collapse, and treatment response	1 *	[49]
	No association between presence of a floppy epiglottis and treatment response	1	[43]
	Absence of complete concentric collapse at the palate was associated with increased treatment response	1	[20]
	Complete collapse at the palate and lateral walls was associated with poorer response compared to patients who had partial collapse at the palate and lateral walls	1 **	[48]
	No association between either palatal or tongue base response during jaw thrust or chin lift and change in AHI	1	[50]
	Lower proportion of antero-posterior or latero-lateral collapse at the palate was associated with increased treatment response	1 *	[49]
	Lower proportion of antero-posterior or latero-lateral collapse at the epiglottis was associated with increased treatment response	1 *	[49]
	A decreased oropharyngeal response during mandibular advancement was associated with increased AHI reduction during HGNS	1 **	[48]
	A decreased palato-oropharyngeal response during mandibular advancement was associated with increased AHI reduction during HGNS	1 **	[48]
	No association between degree of palatal collapse and treatment response	1	[52]
	Higher baseline VOTE-score was associated with increased treatment response	1*	[49]
Endotypes (arousal threshold, muscle responsiveness, loop gain, collapsibility)	Higher arousal threshold was associated with treatment response. Higher muscle responsiveness was associated with treatment response, especially in patients with mild collapsibility. Lower loop gain was associated with treatment response, particularly in patients with milder upper airway collapsibility. Lower collapsibility was associated with treatment failure, specifically in patients with high loop gain and low arousal threshold.	1	[37]
Therapeutic PAP level	Low baseline PAP (<8 cm H_2_O) was associated with AHI reduction and treatment response	1	[38]
SASHB	No association between baseline SASHB and treatment response	1	[37]
**Other**			
Prior upper airway surgery	No association between prior upper airway surgery and treatment outcome	1	[46]
	No association between prior upper airway surgery and treatment outcome	1	[21]

Gray background: Baseline characteristic was the primary outcome in these studies; *, **: Results from the same articles. HGNS (hypoglossal nerve stimulation); AHI (apnea-hypopnea index); BMI (body mass index); ODI (oxygen desaturation index); ESS (Epworth sleepiness scale); PAP (positive airway pressure); SASHB (sleep-apnea specific hypoxic burden).

## Supplementary Materials

The following supporting information can be downloaded at: https://www.mdpi.com/article/10.3390/life14091129/s1, Table S1: Search strategy; Table S2: Extension of study characteristics; Table S3: Associations between age and treatment outcome; Table S4: Associations between sex and treatment outcome; Table S5: Associations between BMI and treatment outcome; Table S6: Associations between AHI and treatment outcome; Table S7: Associations between ODI and treatment outcome; Table S8: Associations between ESS and treatment outcome; Table S9: Associations between neck circumference and treatment outcome; Table S10: Associations between apnea or hypopnea dependency and treatment outcome; Table S11: Associations between oxygen nadir and treatment outcome; Table S12: Associations between site of collapse and treatment outcome; Table S13: Associations between pathophysiological endotyping and treatment outcome; Table S14: Associations between therapeutic PAP level and treatment outcome; Table S15: Associations between SASHB and treatment outcome; Table S16: Associations between prior upper airway surgery and treatment outcome.
