# Supplementary material for: Baseline Characteristics Associated with Hypoglossal Nerve Stimulation Treatment Outcomes in Patients with Obstructive Sleep Apnea: A Systematic Review
Source: Life (Basel). 2024 Sep 7;14(9):1129. doi: 10.3390/life14091129 (PMC11433192; doi:10.3390/life14091129)
Supplement: Supplementary file 1 [file life-14-01129-s001.zip › life-3161802-supplementary.pdf]

Supplementary materials

Article title: Baseline characteristics associated with hypoglossal nerve stimulation treatment outcomes in patients with obstructive sleep apnea: a systematic review
Journal name: Life
Author names: Eldar Tukanov [1,2,*°], Dorine Van Loo[1,2°], Marijke Dieltjens [1,2], Johan Verbraecken [3,4], Olivier M. Vanderveken [1,2,3 §], Sara Op de Beeck [1,2 §]
Affiliations:
[1] : Translational Neurosciences, Faculty of Medicine and Health Sciences, University of Antwerp, 2610 Wilrijk, Belgium
[2] : Department of ENT, Head and Neck Surgery, Antwerp University Hospital, 2650 Edegem, Belgium
[3] : Multidisciplinary Sleep Disorders Centre, Antwerp University Hospital, 2650 Edegem, Belgium
[4] : Research Group LEMP, Faculty of Medicine and Health Sciences, University of Antwerp, 2610 Wilrijk, Belgium
* : Correspondence: eldar.tukanov@uantwerpen.be
° : joint first authorship
§ : joint last authorship

Supplementary methods

Search strategy

Full search strategy used to identify articles. The search had been performed in three different databases.

Supplementary Table S1. Search strategy

Database	Search	Query	Items found
Medline	#1	(obstructi*) AND ((apne*) OR (apno*)) AND ((hypogloss*) OR (tong*) OR (upper airway) OR (upper-airway)) AND (stimulat*) AND ((predict*) OR (effect*) OR (moderator*) OR (associat*) OR (prognos*) OR (measure*))	619
Web of Science	#2	ALL=((obstructi*) AND ((apne*) OR (apno*)) AND ((hypogloss*) OR (tong*) OR (upper airway) OR (upper-airway)) AND (stimulat*) AND ((predict*) OR (effect*) OR (moderator*) OR (associat*) OR (prognos*) OR (measure*)))	801
Cochrane	#3	(obstructi*):ti,ab,kw AND ((apne*) OR (apno*)):ti,ab,kw AND ((hypogloss*) OR (tong*) OR (upper airway) OR (upper-airway)):ti,ab,kw AND (stimulat*):ti,ab,kw AND ((predict*) OR (effect*) OR (moderator*) OR (associat*) OR (prognos*) OR (measure*)):ti,ab,kw	109

ti=title; ab=abstract; kw=keyword; ALL = all fields

Supplementary results

Study characterstics – extended information

Extension on the study characteristics table (Table 1), adding HGNS selection criteria and specific statistical analyses used to assess the association between baseline characteristics and treatment outcome. Baseline characteristics that were the primary outcome of the study have a gray background.

Supplementary Table S2. Extension of study characteristics

First Author	Year	Hypoglossal nerve stimulation selection criteria	Baseline characteristics	Statistical analyses used for association
Bosschieter [39]	2022	ADHERE criteria (see Coca et al. 2022)	AHI (severity-groups)	χ-squared test
Coca [40]	2022	**ADHERE criteria**: AHI 15-65/h; CPAP intolerance; BMI ≤35 kg/m^2; no CCC during DISE	Age, sex, BMI, AHI, ODI, ESS	T-test
Gao [41]	2023	age ≥18 years; AHI 15-65/h; BMI ≤35 kg/m^2; no CCC during DISE; no craniofacial malformations (congenital or acquired); no Down syndrome	Apnea or hypopnea predominancy	Univariable logistic regression
Heiser [23]	2019	ADHERE criteria (see Coca et al. 2022)	Age, sex, BMI, AHI	Uni- and multivariable logistic regression
Kant (a) [42]	2024	AHI 30–50/h or 20–50/h (from August 2021); central AHI ≤25% of total AHI; BMI ≤32 kg/m^2; CPAP intolerance or failure; no CCC during DISE; absence of severe pulmonary, cardiovascular, or psychiatric comorbidities	Site of collapse	Unpaired t-test
Kant (b) [43]	2024	AHI 20–50/h; central AHI ≤25% of total AHI; BMI ≤32 kg/m^2; CPAP intolerance or failure; no CCC during DISE	Site of collapse	Unpaired t-test
Kent [44]	2019	STAR trial criteria (see Ong et al. 2016); GPMS criteria (see Steffen et al. 2018); ADHERE criteria (see Coca et al. 2022)	AHI	Linear regression
			Age, sex, BMI, neck circumference	Linear regression
Kezirian [45]	2014	AHI 20-100; non-REM-AHI ≥15/h; ≥80% hypopneas; central and mixed apnea index <5% of AHI; CPAP intolerance; age 21–70 years; BMI ≤40 kg/m^2 (Australia) or ≤37 kg/m^2 (USA); no prior upper airway surgery, markedly enlarged tonsils, uncontrolled nasal obstruction, severe retrognathia, incompletely treated sleep disorders other than OSA, or major disorder of the pulmonary, cardiac, renal or nervous systems.	BMI	Linear model
Kezirian [46]	2019	ADHERE criteria (see Coca et al. 2022)	Prior upper airway surgery	Logistic (treatment response) and linear (AHI change) regression
Lee [47]	2021	AHI>15; CPAP intolerance; no CCC during DISE.	Age, BMI, AHI	T-test
Lee [38]	2019	AHI > 15 /h; CPAP intolerance; no CCC during DISE	Therapeutic PAP level	χ-squared test
Mulholland [48]	2020	English speaking; AHI ≥15/h; central or mixed AHI ≤25% of total AHI; age ≥22 years; BMI ≤35 kg/m^2; CPAP or MAD intolerance	Site of collapse	Paired t-test
Ong [49]	2016	**STAR trial criteria**: AHI 20-50/h; central and mixed apnea index <25% of AHI; non-supine AHI>10/h; BMI<32 kg/m^2; no CCC during DISE; no grade 3-4 tonsils; no neuromuscular diseases or other significant medical conditions (especially cardiovascular and comorbid non-respiratory sleep disorders)	Age, sex, BMI, AHI, neck size, site of collapse (VOTE)	Independent t-test or Wilcoxon signed rank test
			site of collapse (at specific collapse levels)	χ -squared test
Op de Beeck [37]	2021	STAR trial criteria (see Ong et al. 2016)	Age	Unpaired t-test
			Sex	Fisher's exact test
			BMI, AHI, ODI, SASHB, collapsibility,	Wilcoxon rank-sum test

			muscle responsiveness	
			Arousal threshold, loop gain	Unpaired t-test
			Arousal threshold, collapsibility, muscle responsiveness, loop gain	Multivariable logistic regression
Renslo [50]	2023	N/A	Site of collapse	Uni- and multivariable linear regression
			Age, gender, AHI, oxygen nadir	Uni- and multivariable linear regression
Schwab [51]	2018	Moderate-to-severe OSA (AHI>15/h); BMI<32 kg/m²; no neuromuscular diseases or other significant medical conditions (especially cardiovascular).	Age, BMI, AHI	Nonparametric Wilcoxon rank-sum test
Seay [52]	2020	Age ≥22 years; AHI 15-65/h; CPAP intolerance; no CCC during DISE; central and mixed apnea index <25% of AHI	Age, sex, BMI, AHI, site of collapse	Linear regression
Steffen [21]	2018	**GPMS criteria:** AHI 15-65/h; central AHI ≤25% of total AHI; BMI ≤35 kg/m²; CPAP-intolerance; no CCC during DISE; no tonsil size 3-4; no pregnant patients or planning to become pregnant; no implantable device that may intereact with upper airway stimulation; no requirement of magnet resonance imaging	Age, BMI, AHI, ODI, ESS, neck circumference, prior upper airway surgery	Unpaired t-test
Suurna [53]	2021	ADHERE criteria (see Coca et al. 2022)	BMI	Wilcoxon rank-sum or t-test
Thaler [22]	2020	ADHERE criteria (see Coca et al. 2022)	Age, sex, BMI, AHI	Uni- and multivariable logistic regression
Vanderveken [20]	2013	Moderate-to-severe OSA (AHI≥15/h); BMI<35 kg/m²; no chronic obstructive pulmonary disease, New York Heart Association class III or IV congestive heart failure, neuromuscular diseases, or prior upper airway surgeries not related to OSA	Site of collapse	Wilcoxon signed-rank test
Van de Heyning [54]	2012	AHI ≥25/h; CPAP-intolerance; BMI <35 kg/m²; no chronic obstructive pulmonary disease, New York Heart Association class III or IV congestive heart failure, neuro- muscular diseases, or prior upper airway surgeries not related to OSA	AHI, BMI, ESS	T-test
Wang [55]	2022	AHI 15-65/h; BMI <35 kg/m²; CPAP-intolerance; no CCC during DISE	Age, sex, BMI	Unpaired t-test
Wirth [56]	2022	N/A	AHI	T-test (if normal), Wilcoxon signed-rank test, Mann–Whitney-U test, or Friedman tests
Withrow [57]	2019	ADHERE criteria (see Coca et al. 2022)	Age	T-test
Yu [58]	2021	STAR trial criteria (see Ong et al. 2016)	Age, sex, BMI, AHI, ESS, neck circumference	Parametric t tests or nonparametric Wilcoxon tests for continuous data and χ-squared or Fisher's exact tests for categorical data

Baseline characteristics is the primary outcome of the study when underlined and in bold font. AHI (apnea-hypopnea index); BMI (body mass index); ODI (oxygen desaturation index); ESS (Epworth sleepiness scale); PAP (positive airway pressure); SASHB (sleep-apnea specific hypoxic burden); CPAP (continuous positive airway pressure); MAD (mandibular advancement device; DISE (drug-induced sleep endoscopy); CCC (complete concentric collapse of the palate).

Associations between baseline characteristics and HGNS treatment outcome

Tables each describing a specific baseline characteristic and its associations with HGNS treatment outcome. Results of the statistical tests used to assess these associations are shown as well. Studies for which the baseline characteristic was the primary outcome of the study have a gray background.

Supplementary Table S3. Associations between age and treatment outcome

Age				
**Article**	**Outcome**	**P-value**	**Coefficient**	**OR**
Heiser 2019	Older age was associated with increased treatment success	**0.01**		**1.04 [1.01; 1.08]**
Thaler 2020	Age was not associated with treatment response	>0.05		1.000 [0.976; 1.025]
Withrow 2019	Younger (<65y) and older patients (≥65y) both had a significant AHI reduction when compared with baseline; however, older patients had a larger therapeutic reduction in AHI after 1 year	**0.01**		
Schwab 2018	No difference between responders and non-responders	>0.05		
Lee 2021	No difference between responders and non-responders	>0.05		
Ong 2016	Non-responders were younger	**0.04**		
Op de Beeck 2021	No difference between responders and non-responders	>0.05		
Kent 2019	Younger age was associated with worse improvement in the postoperative AHI		**−0.10 [−0.20; −0.00]**	
Seay 2020	Younger age was not-significantly associated with a favorable treatment response		-4.9 [-14.7; 5.0]	
Coca 2022	No difference between responders and non-responders	>0.05		
Wang 2022	No difference between responders and non-responders	>0.05		
Steffen 2018	No difference between responders and non-responders	0.7955		0.992 [0.932; 1.055]
Renslo 2023	Age was not associated with AHI reduction	>0.05	0.047 [-0.232; 0.326]	
Yu 2021	No difference between responders and non-responders	>0.05		

AHI (apnea-hypopnea index)

Supplementary Table S4. Associations between sex and treatment outcome

Sex				
**Article**	**Outcome**	**P-value**	**Coefficient**	**OR**
Heiser 2019	Sex was not associated with treatment response	>0.05		2.62 [0.88; 7.78]
Thaler 2020	Female sex is associated with favorable treatment response	**0.0008**		**3.363 [1.651; 6.848]**
Ong 2016	No difference between responders and non-responders	>0.05		
Op de Beeck 2021	No difference between responders and non-responders	>0.05		
Kent 2019	No association with AHI reduction		1.93 [−0.84; 4.70]	
Seay 2020	Lower proportion of men in responder group		**−38.9 [−74.2; −3.6]**	
Coca 2022	No difference between responders and non-responders	>0.05		
Wang 2022	No difference between responders and non-responders	>0.05		
Renslo 2023	Male **gender** was associated with reduction in AHI in univariable analysis, but **not in multivariable analysis**	**0.005**	**9.144 [2.803; 15.485]**	
Yu 2021	No difference between responders and non-responders	>0.05		

AHI (apnea-hypopnea index)

Supplementary Table S5. Associations between BMI and treatment outcome

Body mass index (BMI)				
**Article**	**Outcome**	**P-value**	**Coefficient**	**OR**
Suurna 2021	No significant difference in AHI-decrease, between BMI32 (BMI≤32) and BMI35 (32 < BMI ≤ 35) groups	>0.05		
Heiser 2019	Higher BMI was associated with decreased treatment success	**0.03**		**0.91 [0.83; 0.99]**
Thaler 2020	Decrease in BMI is associated with favorable treatment response	**0.0108**		**0.913 [0.851; 0.979]**
Schwab 2018	No difference between responders and non-responders	>0.05		
Lee 2021	No difference between responders and non-responders	>0.05		
Ong 2016	No difference between responders and non-responders	>0.05		
Op de Beeck 2021	Responders showed a statistically, but not clinically, significant difference in baseline BMI (lower BMI)	**0.037**		
Kent 2019	Lower BMI was associated with greater improvement in the postoperative AHI		**0.52; [0.22; 0.83]**	
Seay 2020	Responders had a not statistically significantly lower BMI		1.4 (95% CI, −4.8 to 2.0)	
Kezirian 2014	Subjects with BMI ≤ 35 kg/m² demonstrated a statistically significant AHI reduction.	**<0.001**		
	There were no statistically significant changes in the group with a BMI > 35 kg/m	>0.05		
Coca 2022	Non-responders had a higher BMI	**0.004**		
Van de Heyning 2012	Responders had a statistically significantly lower baseline BMI	**<0.05**		
Wang 2022	Responders had a statistically significantly lower baseline BMI	**0.02**		
Steffen 2018	No difference between responders and non-responders	0.3324		0.921 [0.780; 1.088]
Renslo 2023	Lower BMI was associated with greater improvement in the postoperative AHI	**0.015**	**-0.175 [-1.529; -0.167)**	
Yu 2021	No difference between responders and non-responders	>0.05		

Supplementary Table S6. Associations between AHI and treatment outcome

Apnea-hypopnea index (AHI)				
**Article**	**Outcome**	**P-value**	**Coefficient**	**OR**
Heiser 2019	Baseline AHI was not associated with treatment response	0.88		1.00 [0.98; 1.03]
Thaler 2020	Baseline AHI was not associated with treatment response	0.5198		1.006 [0.988; 1.024]
Bosschieter 2022	No statistically significant difference was found between the subgroups (AHI: 15<30; 30<-50; 50<65; >65) in overall treatment success (66.6%).	0.23		
Schwab 2018	No difference between responders and non-responders	0.087		
Lee 2021	No difference between responders and non-responders	0.83		
Ong 2016	No difference between responders and non-responders	>0.05		
Op de Beeck 2021	No difference between responders and non-responders	>0.05		
Kent 2019	Greater improvement in the postoperative AHI was associated with a higher preoperative AHI		**−0.74 [−0.82; −0.67]**	
Seay 2020	No difference between responders and non-responders		9.3 [−7.3; 25.8]	
Coca 2022	No difference between responders and non-responders	>0.05		
Van de Heyning 2012	Responders had a statistically significantly lower baseline AHI	**<0.01**		
Wirth 2022	No difference between responders and non-responders	0.106		

Steffen 2018	No difference between responders and non-responders	0.0854		0.961 [0.919; 1.006]
Renslo 2023	Higher preoperative AHI was associated with greater improvement in the postoperative AHI	**<0.001**	**0.758 [0.737; 1.093]**	
Yu 2021	No difference between responders and non-responders	>0.05		

Supplementary Table S7. Associations between ODI and treatment outcome

Oxygen desaturation index (ODI)				
**Article**	**Outcome**	**P-value**	**Coefficient**	**OR**
Op de Beeck 2021	No difference between responders and non-responders	>0.05		
Coca 2022	No difference between responders and non-responders	>0.05		
Steffen 2018	Responders had a lower baseline ODI	**0.0434**		**0.962 [0.926; 0.999]**

Supplementary Table S8. Associations between ESS and treatment outcome

Epworth sleepiness scale (ESS)				
**Article**	**Outcome**	**P-value**	**Coefficient**	**OR**
Seay 2020	No difference in ESS between responders and non-responders		−4.2 [−8.8; 0.4]	
Coca 2022	No difference in ESS between responders and non-responders	>0.05		
Van de Heyning 2012	No difference in ESS between responders and non-responders	>0.05		
Steffen 2018	No difference in ESS between responders and non-responders	>0.05		
Yu 2021	No difference in ESS between responders and non-responders	>0.05		

Supplementary Table S9. Associations between neck circumference and treatment outcome

Neck circumference				
**Article**	**Outcome**	**P-value**	**Coefficient**	**OR**
Ong 2016	No difference in neck size between responders and non-responders	>0.05		
Kent 2019	No association between neck circumference and AHI		1.73 [−1.62; 5.07]	
Steffen 2018	No difference in neck circumference between responders and non-responders	>0.05		
Yu 2021	No difference in neck circumference between responders and non-responders	>0.05		

AHI (apnea-hypopnea index)

Supplementary Table S10. Associations between apnea or hypopnea dependency and treatment outcome

Apnea- or hypopnea dependency				
**Article**	**Outcome**	**P-value**	**Coefficient**	**OR**
Gao 2023	No difference in meeting treatment response between apnea- and hypopnea-predominant groups	0.14		3.37 [0.78; 23.52]

Supplementary Table S11. Associations between oxygen nadir and treatment outcome

Oxygen nadir				
**Article**	**Outcome**	**P-value**	**Coefficient**	**OR**
Renslo 2023	On univariable analysis, preoperative oxygen nadir was not a significant predictor of AHI change	0.13	-0.377 [-0.870; 0.116]	
	On multivariable analysis preoperative oxygen nadir was a significant predictor of AHI change	**0.008**	**0.202 [0.151; 0.970]**	

AHI (apnea-hypopnea index)

Supplementary Table S12. Associations between site of collapse and treatment outcome

Site of collapse				
**Article**	**Outcome**	**P-value**	**Coefficient**	**OR**
**Ong 2016**	Non-responders had a higher baseline VOTE score (calculated as the sum of the collapse degree (2 for complete, 1 for partial, and 0 for no collapse) at each site of obstruction for a maximum score of 8)	**0.02**		
	Non-responders had a higher proportion of complete AP or LL collapse at the velum compared with therapy responders.	**0.01**		
	Non-responders had a higher proportion of complete AP or LL collapse at the epiglottis compared with therapy responders.	**0.01**		
	No difference in degree of oropharynx or tongue base collapse between responders and non-responders	>0.05		
**Vanderveken 2013**	Treatment success in the subset of patients without CCC collapse at the level of the palate was 81% (13/16), while treatment success could not be achieved in any patient with CCC collapse at the level of the palate in this study (0/5).			
	Patients with palatal CCC did not have a significant change in AHI with UAS 6 months after implantation. The patients without palatal CCC had a statistically significant improvement in AHI with UAS despite multilevel collapse at the palate and tongue base			
**Renslo 2023**	No association between palatal response during jaw thrust and change in AHI.	0.21	4.059 [-2.307; 10.424]	
	No association between palatal response during chin lift and change in AHI.	0.63	-2.524 [-12.766; 7.718]	
	No association between tongue base response during jaw thrust and change in AHI.	0.24	-5.301 [-14.092; 3.490]	
	No association between tongue base response during chin lift and change in AHI.	0.24	3.768 [-2.552; 10.087]	
Mulholland 2020	Patients with a minimal opening of velar and oropharyngeal collapse during mandibular advancement (n = 36) had a greater mean decrease in AHI of 21.1 compared to patients with a robust opening of velar and oropharyngeal collapse during mandibular advancement (n = 10), with a mean decrease in AHI of 4.9 (P = .02).	**0.02**		
	Patients with decreased opening of the lateral wall in response to mandibular advancement (n = 40) demonstrated greater AHI improvement than those with increased opening (>90% collapse to <50% collapse) (n = 6), mean AHI reduction of 21.0 versus 0.2.	**0.03**		
	Patients who had complete baseline collapse at the palate and lateral walls (n = 11) had poorer response compared to patients who had partial collapse of the soft palate and lateral walls (n = 35), AHI reduction of 5.6 versus 22.3	**0.02**		
	Patients with partial baseline collapse of the lateral walls (n = 32) had larger AHI reductions compared to those with complete collapse (n = 14), AHI reduction of 22.4 versus 9.0. (not significant)	0.05		
Kant 2024 (a)	Both the group with complete AP palatal collapse and the group with upper palatal AP and lower palatal CCC collapse had a statistically significant AHI reduction	**<0.05**		
	No difference in response rates between the group with complete AP palatal collapse and group with upper palatal AP and lower palatal CCC collapse.	>0.05		
Kant 2024 (b)	No difference in therapeutic response between groups with and without floppy epiglottis	0.659		
Seay 2020	No difference in percent collapse at the velum between responders and nonresponders.		−7.8 [−22.0; 6.4]	

AHI (apnea-hypopnea index); CCC (complete concentric collapse of the palate); AP (anteroposterior); LL (laterolateral); VOTE (velum-oropharynx-tongue base-epiglottis)

Supplementary Table S13. Associations between pathophysiological endotyping and treatment outcome

Pathophysiological endotyping				
**Article**	**Outcome**	**P-value**	**Coefficient**	**OR**
Op de Beeck 2021	Responders showed a significantly higher baseline arousal threshold compared to non-responders	**0.044**		
	A high arousal threshold at baseline was associated with greater likelihood of HGNS response, independent of AHI, collapsibility, and other traits	**0.001**		**6.76 [2.44; 23.3]**
	Collapsibility was more pronounced in responders versus nonresponders	**0.009**		
	Greater collapsibility promotes HGNS response in patients with non-anatomical deficits (namely, higher loop gain and lower arousal threshold)	0.060		0.51 [0.24; 1.00]
	There was no statistically significant difference in muscle responsiveness at baseline between responders and non-responders	0.65		
	Greater muscle responsiveness promotes success, especially in patients with mild collapsibility	**0.004**		**4.22 [1.70; 12.55]**
	There was no statistically significant difference in loop-gain at baseline between responders and non-responders	0.85		
	Lower loop gain was associated with better responses to HGNS, particularly in patients with milder upper airway collapsibility	0.056		**0.50 [0.23–0.98]**

HGNS (hypoglossal nerve stimulation); AHI (apnea-hypopnea index)

Supplementary Table S14. Associations between therapeutic PAP level and treatment outcome

Therapeutic positive airway pressure (PAP) level				
**Article**	**Outcome**	**P-value**	**Coefficient**	**OR**
Lee 2019	The low PAP group (<8cm H_2O) achieved a significantly larger AHI reduction than the high PAP group (≥8cm H_2O)	**0.02**		
	The low PAP group (<8cm H_2O) achieved a significantly higher treatment response than the high PAP group (≥8cm H_2O)	**<0.01**		

Supplementary Table S15. Associations between SASHB and treatment outcome

Sleep-apnea specific hypoxic burden (SASHB)				
**Article**	**Outcome**	**P-value**	**Coefficient**	**OR**
Op de Beeck 2021	No difference in sleep apnea-specific hypoxic burden between responders and non-responders	>0.05		

Supplementary Table S16. Associations between prior upper airway surgery and treatment outcome

Prior upper airway surgery				
**Article**	**Outcome**	**P-value**	**Coefficient**	**OR**
Kezirian 2019	Any previous surgery and the subgroups of previous palate surgery and previous hypopharyngeal surgery were not associated with treatment response or change in AHI	>0.05 for all variables		All not significant
Steffen 2018	No difference in prior upper airway surgery between responders and non-responders	>0.05		

AHI (apnea-hypopnea index)
